# Mice Susceptible to SARS Coronavirus

**DOI:** 10.3201/eid1007.031119

**Published:** 2004-07

**Authors:** David E. Wentworth, Laura Gillim-Ross, Noel Espina, Kristen A. Bernard

**Affiliations:** * New York Department of Health, Albany, New York, USA;; † State University of New York, Albany, New York, USA

**Keywords:** SARS-CoV, Coronavirus, SARS Virus, mice, model, multiplex, RT-PCR, PCR, dispatch

## Abstract

Murine models of severe acute respiratory syndrome–associated coronavirus (SARS-CoV) will greatly advance research on this emerging virus. When BALB/c mice were simultaneously inoculated intranasally and orally, replication of SARS-CoV was found in both lung and intestinal tissue.

The outbreak of severe acute respiratory syndrome (SARS) that emerged in China in November 2002 was caused by a novel coronavirus (SARS-CoV) that was detected in lungs, nasopharyngeal aspirates, and feces of infected patients (1–4). This outbreak in humans is striking because of the high rate of illness and death associated with it. The SARS-CoV outbreak likely resulted from zoonotic transmission, and natural animal reservoirs of viruses nearly identical to SARS-CoV increase the likelihood of its reemergence in humans (5).

Coronaviruses are enveloped, plus-stranded RNA viruses that cause important respiratory and enteric diseases of humans and many animal species. Large peplomers or spike glycoproteins (S) are the viral attachment proteins that protrude from the virion and give it the appearance of a corona. Coronaviruses are members of the *Nidovirales*, which produce 3´ co-terminal nested subgenomic mRNAs upon entry into susceptible host cells. The genome is the largest of all RNA viruses (27.6–31.2 kb), and the genomic RNA is infectious when transfected into cells from a wide variety of species and tissue types. Yet most strains of coronavirus have very restricted species and tissue tropism, illustrating the major role S-receptor interactions play in the species specificity and pathogenesis of coronaviruses. SARS-CoV sequence analysis shows that it has many of the unique characteristics of coronaviruses and that it shares the most predicted amino acid similarity and other molecular signatures with serogroup 2 coronaviruses (6).

Animal models of SARS-CoV are important for the study of virus-host interactions. Cats, ferrets, and nonhuman primates have been experimentally infected with SARS-CoV (7,8). In addition, SARS-CoV–like viruses were isolated from palm civet cats and closely related raccoon dogs, which are sold in markets in China (5). All of these animal species are important for the in vivo study of SARS-CoV. However, a murine model is also necessary to evaluate antiviral agents, vaccines, and immune response. Previous studies in suckling mice inoculated intracranially or intraperitoneally suggest that mice are not permissive to SARS-CoV (1,8). On the other hand, the infection of divergent species suggests that many animal species may be susceptible (5,8).

Human and animal coronaviruses are transmitted by the respiratory or enteric routes and initially infect epithelial cells of these tissues (9). Thus, a combined intranasal and oral injection of mice was explored as a potential animal model for SARS-CoV. Four-week-old, female BALB/c mice were inoculated intranasally and orally with 2 x 10^5^ 50% tissue culture infective dose of SARS-CoV Urbani or were mock-inoculated with carrier alone. Mice were weighed and observed for clinical signs daily throughout the study. Three SARS-CoV–inoculated and one mock-inoculated mouse were euthanized 3, 5, 7, 10, and 28 days postinoculation (p.i.). Tissues harvested on euthanasia included blood, lungs, and small intestine (ileum). Total RNA was isolated from the lungs and intestines. All work with mice was conducted at the Wadsworth Center, New York Department of Health, Albany, under a protocol approved by the Institutional Animal Care and Use Committee. All experiments with infectious SARS-CoV were performed in a biosafety level 3 laboratory and were conducted under appropriate conditions, with precautions that adhered to, or exceeded, the requirements set forth in "Interim Laboratory Biosafety Guidelines for Handling and Processing Specimens Associated with SARS" (available from www.cdc.gov/ncidod/sars/sarslabguide.htm).

To specifically identify virus replication, a multiplex reverse transcriptase–polymerase chain reaction (RT-PCR) was used to simultaneously amplify glyceraldehyde 3 phosphate dehydrogenase (G3PDH), SARS-CoV genomic RNA (gRNA), and subgenomic RNA (sgRNA) (10). Upon entry into host cells the coronavirus gRNA (27–31.5 kb) serves as an mRNA to translate two large polyproteins (1a and 1ab). The polyproteins are autocatalytically processed into replicative enzymes, including the RNA-dependent RNA polymerase, which synthesizes both negative-sense and positive-sense sgRNAs, and the positive-sense sgRNAs serve as mRNAs for all of the open reading frames (ORFs) downstream of ORF1b (6,11). SARS-CoV infects Vero, Vero E6, and primary rhesus monkey kidney cells in culture, and infected cells have a nested set of eight 3´ co-terminal of mRNAs, each of which has at its 5´ end a leader sequence derived from the 5´ terminus of the genome (6,12). We took advantage of the unique features of CoV replication, sgRNA transcription in particular, to develop multiplex primers to differentiate input gRNA from sgRNA that is produced upon entry into the host cell. A 180-bp amplicon is produced from a sense primer (SARS-1 [5´-ATATTAGGTTTTTACCTACCCAGG-3´]) that is specific for the leader sequence and an antisense primer specific for the SARS-CoV spike glycoprotein gene (SARS-21,593R [5´-AGTATGTTGAGTGTAATTAGGAG-3´]). This amplicon is produced only when sgRNA is present and indicates virus entry and replication. To identify gRNA (input and newly synthesized), a sense primer that binds the 3´ terminus of the 1b gene (SARS-21,263 [5´- TGCTAACTACATTTTCTGGAGG-3´] was paired with SARS-21,593R to produce a 276-bp amplicon. The amplification was performed as a multiplex reaction for SARS-CoV gRNA, sgRNA, and G3PDH by using a OneStep RT-PCR procedure (Qiagen, Inc., Valencia, CA); thus, both positive- and negative-sense SARS-CoV RNAs served as templates for reverse transcription.

Production and persistence of gRNA and sgRNA were examined in permissive and nonpermissive cells. Analysis of permissive Vero E6 cells showed that the sgRNA encoding S was not present in the input virus before RNA replication (Figure 1A, 1 h). However, S sgRNAs produced after entry were detected at 16 h and 5 d after SARS-CoV inoculation. Additionally, gRNA qualitatively increased after entry of the virus. In contrast, sgRNA was not detected in nonpermissive murine (L2) or human (MRC5) cell lines, and gRNA fell below detectable limits by 5 d. The reaction conditions were optimized to favor amplification of SARS-CoV gRNA and sgRNA; thus, when they were present at high levels, amplification of G3PDH was reduced. Amplification of G3PDH was used to demonstrate RNA integrity, and it was always detected in the absence of viral RNAs.

**Figure 1:**
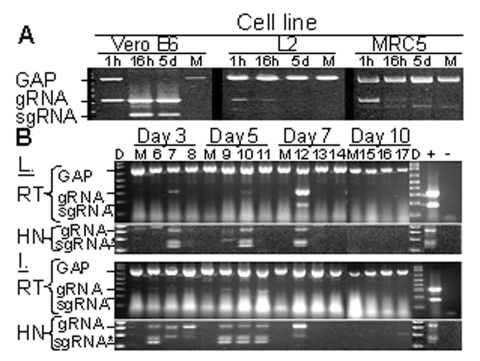
Replication-specific multiplex reverse transcriptase–polymerase chain reaction (RT-PCR) assay shows severe acute respiratory syndrome–associated coronavirus (SARS-CoV) replicated in the lungs and intestines of mice. A) Vero E6, murine fibroblast (L2), and human lung fibroblasts (MRC5) were inoculated with SARS-CoV at an MOI of ≈0.001 or were mock-inoculated (M). G3PDH, SARS-CoV gRNA, and sgRNA were amplified by multiplex RT-PCR from total RNA extracted at 1 h, 16 h, or 5 days after inoculation. Amplicons were visualized by ethidium bromide staining after electrophoresis; negative images are shown. B) Mice were inoculated with 2x10^5^ 50% tissue culture infective dose of SARS-CoV (lanes labeled 6–17) or were mock-inoculated (M) and euthanized after 3, 5, 7, or 10 days. G3PDH, SARS-CoV gRNA, and sgRNA were amplified by multiplex RT-PCR from total RNA extracted from the lung (L) and intestine (I) harvested at various time points. Heminested PCR (HN) was used to amplify gRNA and sgRNA from RT-PCR reactions. Positive and negative controls for PCR reactions are indicated by + and –, respectively. D indicates DNA marker ladder. *The doublet observed in HN-PCR reactions results from residual primers used in the primary amplification reaction.

Lungs from the experimentally inoculated mice were analyzed by the multiplex assay. One of three mice from each group sacrificed on days 3, 5, and 7 p.i. showed evidence of lung infection (Figure 1B). The presence of gRNA on days 5 and 7 is strong evidence for viral replication since the inoculum is most likely degraded, as is seen with nonpermissive cells in vitro (Figure 1A). In addition, sgRNA, which is indicative of virus replication, was amplified from the lung RNA of a mouse on day 7 p.i. (Figure 1B, sgRNA). Heminested PCR of the first-round multiplex RT-PCR amplicons showed that SARS-CoV gRNA and sgRNA were present in the lungs of each of these mice. Another animal euthanized 5 days p.i. also showed evidence of infection by the heminested PCR (Figure 1B, HN). SARS-CoV gRNA or sgRNA was not detected in the lungs of mice 10 days p.i. or in any mock-inoculated animals (Figure 1). Infection of lung tissue in mice is consistent with the tropism of SARS-CoV in humans and experimentally inoculated nonhuman primates, ferrets, and cats (4,7,8).

Coronaviruses of many animal species, including porcine, feline, canine, murine, and bovine, infect intestinal tissue (9). In humans, SARS-CoV causes interstitial pneumonia with fever and sometimes diarrhea (3,4). In our study, SARS-CoV gRNA was present in the intestines of all virus-inoculated mice at 3 and 5 days p.i. (Figure 1B). Heminested PCR of these amplicons showed that sgRNA was also present in all virus-inoculated animals on days 3 and 5. One mouse had sgRNA in the ileum 7 days p.i.; this same mouse that had qualitatively high levels of gRNA and sgRNA in the lung (mouse 12). Identification of SARS-CoV replication in the small intestines of mice is consistent with the enteric disease observed in some human SARS-CoV infections and with the identification of SARS-CoV gRNA in the stomach and duodenum of an experimentally infected cynomolgus macaque (4,7).

The mice were assessed for clinical disease and weight loss. Subtle clinical disease was observed in some of the mice; four mice had ruffled fur for >3 days, including mouse 12, which had qualitatively high levels of gRNA and sgRNA in its lung (Figure 1B). No respiratory distress or diarrhea was observed throughout the study. The virus-inoculated mice tended to gain less weight than the mock-inoculated mice (Figure 2A). In addition, 3 of 15 mice lost 5%–6% body weight 3 days p.i., and 1 of 9 mice lost 6% body weight on day 7. Overall, six mice exhibited either mild clinical signs or weight loss throughout the study, while mock-inoculated mice remained unaffected. This finding suggests that SARS-CoV caused a subclinical infection or a very mild disease in mice.

**Figure 2:**
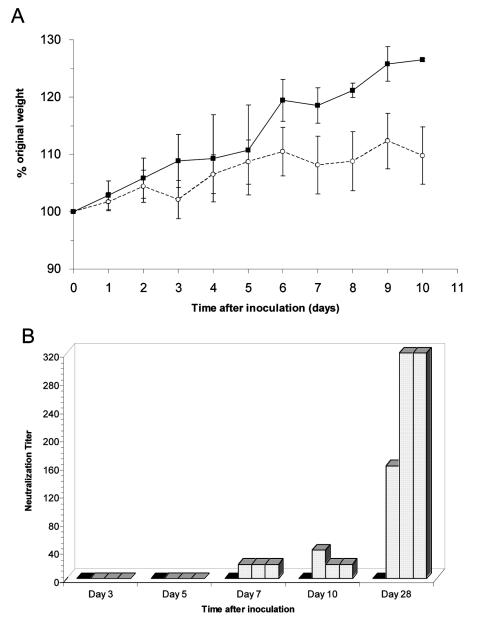
Mice inoculated with severe acute respiratory syndrome–associated coronavirus showed decreased weight gain and developed neutralizing antibodies. A) Average percentage original weight for 2 to 5 mock-inoculated (gray line and solid squares) and 6 to 15 virus-inoculated (solid line and open circles) mice. Error bars represent 1 standard deviation. B) Neutralization titers reported as reciprocal of serum dilution for individual mock-inoculated (solid bars) and virus-inoculated (bars with hatched marks) mice at time of sacrifice. Lowest dilution tested was 1:20.

Serum antibody to SARS-CoV was analyzed by a virus neutralization assay (Figure 2B). Mock-inoculated mice showed no virus neutralization, nor was neutralizing antibody detected in mice euthanized 3 or 5 days p.i. Neutralizing antibodies were detected in all mice sacrificed 7, 10, and 28 days p.i., and the titers were 8- to 16-fold higher on day 28. These neutralizing titers are similar to those reported for naturally and experimentally infected animals (5,8). In addition, seroconversion to SARS-CoV is the accepted standard for the determination of human infections by SARS-CoV and continues to be more reliable than RT-PCR methods (4).

The presence of gRNA and, more importantly, sgRNA in the lungs and intestines shows that SARS-CoV replicated in these tissues. Furthermore, the high neutralizing antibody titers on day 28 p.i. are supportive of an active viral infection. The presence of SARS-CoV RNAs or neutralizing antibodies demonstrates that all 15 inoculated mice were infected. The results of this study suggest that SARS-CoV peaks early (days 3–5), and the immune response clears the virus from the lung and intestine by 10 days, which suggsts that viral clearance in mice is more rapid than in human patients, who begin to recover 7–12 days after the onset of clinical illness (approximately 9–14 days after infection) (3,4). In 10% to 15% of patients, the initial phase of disease is followed by more severe pulmonary disease characterized by respiratory distress, pulmonary infiltration of mononuclear inflammatory cells, multinucleated syncytia, and fibrosis (3,4). The pathophysiology of the late complications of SARS is not understood, but immunopathology could play a critical role in the disease. This study opens many potential avenues of research using wild-type, transgenic, or knockout mice to answer questions of how age, sex, prior exposure, and immune response influence the pathogenesis of SARS-CoV.
